# A non-randomised controlled pilot study of clinical pharmacist collaborative intervention for community dwelling patients with COPD

**DOI:** 10.1038/s41533-018-0105-7

**Published:** 2018-10-10

**Authors:** Vicki Hunt, Dave Anderson, Richard Lowrie, Colette Montgomery Sardar, Susan Ballantyne, Graeme Bryson, John Kyle, Peter Hanlon

**Affiliations:** 1 East Renfrewshire Health and Social Care Partnership Eastwood Health and Care Centre, Drumby Crescent, Glasgow, Scotland United Kingdom; 2 The New Victoria Infirmary, Glasgow, United Kingdom; 3 Pharmacy and Prescribing Support Unit, NHS Greater Glasgow and Clyde, Glasgow, G3 8SJ United Kingdom; 4 Prescribing Support Pharmacy Team, North East Glasgow HSCP, Glasgow, United Kingdom; 5 Glasgow City Health and Social Care Partnership, Glasgow, United Kingdom; 6 General Practice and Primary Care Institute of Health and Wellbeing, University of Glasgow, Glasgow, United Kingdom

## Abstract

UK, home-based patients with COPD receive specialist care from respiratory physicians, nurses, and general practitioners (GPs), but increasing complexity of therapeutic options and a GP/Nurse workforce crisis suggests merit in testing the role of home visits by a clinical pharmacist. We conducted a non-randomised intervention study with a contemporaneous comparator group, in Glasgow (Scotland). A clinical pharmacist (working closely with a consultant respiratory physician) visited patients with COPD living at home, assessing respiratory and other co-morbid conditions, and medicines then, with patient approval, agreed treatment modifications with a consultant physician. Comparator group-patients were drawn from another hospital out-patient clinic. Main outcomes were exacerbations during 4-months of follow-up and respiratory hospitalisations (number and duration) after 1 year. In the intervention group, 86 patients received a median of three home visits; 87 received usual care (UC). At baseline, patients in the intervention group were similar to those in UC in terms of respiratory hospitalisations although slightly younger, more likely to receive specific maintenance antibiotics/Prednisolone and to have had exacerbations. Sixty-two (72.1%) of the intervention group received dose changes; 45 (52.3%) had medicines stopped/started and 21 (24.4%) received an expedited review at the specialist respiratory consultant clinic; 46 (53.5%) were referred to other healthcare services. Over one-third were referred for bone scans and 11% received additional investigations. At follow-up, 54 (63.5%) of intervention group participants had an exacerbation compared with 75 (86.2%) in the UC group (*p* = 0.001); fewer had respiratory hospitalisations (39 (45.3%) vs. 66 (76.7%); *p* < 0.001). Hospitalisations were shorter in the intervention group. Pharmacist-consultant care for community dwelling patients with COPD, changed clinical management and improved outcomes. A randomised controlled trial would establish causality.

## COPD: At-home pharmacist visits improve health outcomes

Clinical pharmacists, working in collaboration with respiratory specialists, can help people who live at home with chronic obstructive pulmonary disease (COPD) better manage their medications and symptoms. In a non-randomised pilot study, Richard Lowrie from the NHS Greater Glasgow and Clyde, UK, and colleagues found that patients with COPD who receive standard at-home care—which includes visits to GP surgeries and hospital-based respiratory out-patient clinics, and visits from respiratory specialist nurses—were more likely to experience exacerbations and need lengthy hospital stays than those who additionally received home visits from a clinical pharmacist. The pharmacist, in consultation with the patient’s respiratory physician, often proposed medication changes and suggested additional testing or referrals that presumably explain the improved health outcomes. The authors conclude that a large, randomised trial is warranted to further evaluate the merits of this collaborative intervention for community dwelling patients with COPD.

## Introduction

Chronic obstructive pulmonary disease (COPD) is a treatable disease characterised by progressive airflow obstruction in response to noxious particles or gases in the airways and lung, and is the fourth leading cause of death worldwide.^1^ Smoking is the predominant causative factor, with other exposures (e.g., occupational) also contributing, and COPD prevalence is projected to increase.^2,3^ Advancing disease is characterised by recurrent exacerbations (acute worsening of symptoms requiring additional therapy), which often require hospital admission.^2^ Co-morbidity is common and impacts negatively on quality of life and mortality.^4^ The economic burden of COPD is considerable, estimated at €38.6 billion per year (56% of the cost of respiratory disease) in the European Union alone with a significant proportion resulting from unscheduled healthcare use resulting from exacerbations.^5^ COPD admissions follow a socioeconomic gradient with higher rates in patients living in poor areas.^6^ Optimal pharmacological treatment including rapid treatment of exacerbations, can improve symptoms, reduce exacerbation frequency, and improve exercise tolerance^2,7^ while poor medication adherence^8^ and suboptimal inhaler technique negatively impact outcomes.^2,9–11^ The association between poor medication adherence, and morbidity and mortality in COPD, together with the preventable nature of some exacerbations through tailored use of medicines,^12,13^ suggests a potential role for pharmacists. Community Pharmacists and clinical pharmacists working in hospitals, by improving adherence, inhalation technique, and encouraging smoking cessation, may decrease exacerbation rates.^14–18^ Review and modification of medicines for COPD and other morbidities by pharmacists has not been tested, as far as we are aware.

In the UK, backed by strategic policy and a shortage of general practitioners (GPs) and Nurses, Primary Care-based clinical pharmacists work in general practices to support prescribing.^19,20^ Since the first study of clinical pharmacists reviewing elderly patients with multiple morbidities receiving polypharmacy in Glasgow general practices in 1997,^21,22^ research has informed expanded roles to include patients with heart failure and multimorbidities^23^ and on prescribing through educational outreach targeted at GPs and nurses in general practices.^24^ To our knowledge, only one UK-based study from over 10 years ago, showed an out-patient pharmacist-led intervention focussing only on COPD improved outcomes in COPD.^15^ A more recent UK study using pre- and post-intervention comparison, targeted patients with COPD presenting to community pharmacies. Pharmacists delivered a multifaceted intervention including therapy optimisation, however, they did not access medical records or collaborate with other members of the multidisciplinary team, which limited the scope for introduction of changes. Adherence and quality of life improved while GP visits declined following pharmacist advice. The authors suggested the intervention may have been cost effective.^25^ However, home visits by a clinical pharmacist to review and change medicines for COPD and other co-morbid conditions, working collaboratively with a consultant respiratory physician for respiratory issues, remains untested, despite the growing disease burden, and the potential for improvements in management.^26^

In Scotland, among patients with long-term conditions, rates of non-attendance at general practices was highest (12%) among people with COPD,^27^ and non-attendance was higher among patients living in more socioeconomically deprived areas^27^ suggesting the need for complementary, additional models of care at home for this group of patients. Non-attendance at general practices is associated with increased mortality.^28^ Across the UK, respiratory nurses treat patients at home following assessment in a hospital respiratory unit,^29^ and domiciliary support from specialist respiratory nurses may be provided following respiratory hospitalisation, with patients receiving out-patient physician, and physiotherapy follow-up in hospital-based clinics.^30^ However, other than during visits to their GP, patients do not receive a review of all of their conditions together despite the prevalence and negative impact of co-morbidities in COPD.^31^

Recent calls for more community-based models of care to help minimise unscheduled healthcare use and reduce costs^32^ raises the question: can a general practice-based clinical pharmacist—consultant physician pilot intervention improve outcomes in patients with moderate or severe COPD, through home visits? We aimed to evaluate the impact of pharmacist-consultant respiratory physician intervention on exacerbations, and respiratory hospitalisations. The intervention involves a pharmacist visiting patients with COPD in their homes to assess respiratory symptoms and other co-morbid conditions then introduce changes (improvements) in the management of respiratory and other co-morbid conditions, following discussion with the patient’s respiratory physician. Changes introduced by the pharmacist included prescribing of medicines, monitoring, or onward referral, e.g., for bone density measurement. Patients were visited several times, and the pharmacist communicated all changes to the patient’s GP. This paper examines findings, from a non-randomised controlled pilot study.

## Results

### Patient identification and engagement

In the intervention group, 88 patients were asked to participate; 86 agreed and two declined. The pharmacist visited patients for the first time at home from 5th March 2015 to 16th February 2016. The last follow-up visit occurred on 31st March 2016. Baseline data on exacerbations (acute prescribing of antibiotics or oral steroids) were collected in the 4-month period leading up to the date of first consultation for each of the 86 patients who received a first visit and follow-up data on exacerbations were collected in the 4-month (112 days) period following the date of first visit (Table 1).

**Table 1:** Intervention group: months included in exacerbations

Month	No. of patients *n* = 86	Months included^a^ in baseline (exacerbations)	Months included^a^ in follow-up (exacerbations)
March 2015	13	Nov^a^, Dec, Jan, Feb, Mar^a^	March^a^, Apr, May, Jun, Jul^a^
Apr 2015	10	Dec^a^, Jan, Feb, Mar, Apr^a^	Apr^a^, May, Jun, Jul, Aug^a^
May 2015	6	Jan^a^, Feb, Mar, Apr, May^a^	May^a^, Jun, Jul, Aug, Sep^a^
Jun 2015	8	Feb^a^, Mar, Apr, May, Jun^a^	Jun^a^, July, Aug, Sep, Oct^a^
Jul 2015	2	March^a^, Apr, May, Jun, Jul^a^	Jul^a^, Aug, Sep, Oct, Nov^a^
Aug 2015^b^	12	Apr^a^, May, Jun, Jul, Aug^a^	Aug^a^, Sep, Oct, Nov, Dec^a^
Sep 2015	9	May^a^, Jun, Jul, Aug, Sep^a^	Sep^a^, Oct, Nov, Dec, Jan^a^
Oct 2015	7	Jun^a^, July, Aug, Sep, Oct^a^	Oct^a^, Nov, Dec, Jan, Feb^a^
Nov 2015	5	Jul^a^, Aug, Sep, Oct, Nov^a^	Nov^a^, Dec, Jan, Feb, Mar^a^
Dec 2015	4	Aug^a^, Sep, Oct, Nov, Dec^a^	Dec^a^, Jan, Feb, Mar, Apr^a^
Jan 2016	6	Sep^a^, Oct, Nov, Dec, Jan^a^	Jan^a^, Feb, Mar, Apr, May^a^
Feb 2016	4	Oct^a^, Nov, Dec, Jan, Feb^a^	Feb^a^, Mar, Apr, May, Jun^a^

^a^Part month

^b^Baseline and follow-up data collection in comparator group

Eighty-seven patients were included in the comparator group, representing all eligible patients on the respiratory nurse list of Queen Elizabeth University Hospital on 5th March 2015. All 87 patients in the comparator group had baseline exacerbation data collected from the 4-month period leading up to 21st August 2015, and follow-up exacerbations data extracted from the 4-month period from 1st November 2015–28th February 2016. Forty-nine (57%) of patients in the intervention group seen for the first time from July 2015 through February 2016, had follow-up months that overlapped with the period covered in follow-up of patients in the comparator group (Table 1).

**Fig. 1:**
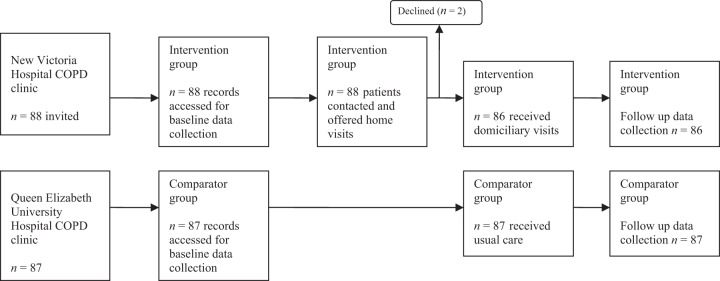
Flow of participants through the study

Figure 1 describes patient flow through the study.

In the intervention group, following the initial visit, nine patients did not have any subsequent visits: the pharmacist did not identify a need to follow-up five patients; one patient was hospitalised; one patient was lost to follow-up and two patients died. The remaining 77/86 (90%) received subsequent visits: the median number of follow-up contacts per patient was 3 (range 1 to 10) and the median duration of intervention was 16 weeks (3 to 44 weeks). Duration of domiciliary visits averaged 30 min (10 to 90 min) and the approximate duration of weekly discussions between consultant and pharmacist was 1 h. On average, the hour spent with the consultant per week enabled discussion of four or five patients.

Table 2 shows demographic and clinical variables at baseline.

**Table 2:** Baseline characteristics of patients

Characteristic^a^	Intervention group *n* = 88	Comparator group *n* = 87	*p-*value
Demographics			
Mean age, years (SD)	67.9 (9.8)	72.1 (9.4)	0.005
Gender, % female	67.1%	66.7%	0.958
Ethnicity (% white British)	88 (100%)	87 (100%)	1.00
BMI (kg/m^2^; SD)^b^	25.5 (6.1)	26.3 (6.8)	0.430
Socioeconomic deprivation^c^			
1 (most deprived)	45 (52.9%)	41 (47.1%)	0.086
2	16 (18.8%)	19 (21.8%)	
3	4 (4.7%)	13 (14.9%)	
4	7 (8.2%)	8 (9.2%)	
5 (least deprived)	13 (15.3%)	6 (6.9%)	
Clinical			
Smoking (patient reported)			
Never smoked	2 (2.3%)	1 (1.2%)	0.675
Current smoker	23 (26.1%)	27 (31.0%)	
Ex-smoker	63 (71.6%)	59 (67.8%)	
% predicted FEV_1_ (mean +/− SD)	48.5 (18.3)	52.5 (19.3)	0.194
FEV_1_ Stage^h^			
Mild (≥80% predicted)	6 (7.1%)	8 (10.7%)	0.088
Moderate (50–79%)	28 (33.3%)	27 (36.0%)	
Severe (30–49%)	37 (44.0%)	37 (49.3%)	
Very severe (<30%)	13 (15.5%)	3 (4.0%)	
COPD assessment test (CAT) (median (IQR)^d^	27 (22–31)	–	–
Long-term oxygen therapy	28 (31.8%)	22 (25.3%)	0.339
Medical Research Council (MRC) score^e^			
1	0 (0%)	1 (1.4%)	0.070
2	3 (3.5%)	3 (4.4%)	
3	6 (6.9%)	7 (10.1%)	
4	33 (37.9%)	31 (45.0%)	
5	45 (51.7%)	27 (39.0%)	
Number of co-morbidities (median (IQR)	3 (2–4)	4 (2–6)	0.008
Type of co-morbidity			
Asthma	12 (14.0%)	11 (12.6%)	0.80
Other respiratory co-morbidity (not COPD or asthma)	20 (23.3%)	15 (17.2%)	0.32
Lung cancer	1 (1.2%)	5 (5.7%)	0.10
Any cancer	18 (20.9%)	15 (17.2%)	0.78
Occlusive vascular disease	19 (22.1%)	25 (28.7%)	0.75
Cardiovascular disease	41 (47.7%)	51 (58.6%)	0.66
Gastrointestinal disease	27 (31.4%)	29 (33.3%)	0.63
Central Nervous System	31 (36.0%)	33 (37.9%)	0.91
Osteoporosis	14 (16.3%)	19 (21.8%)	0.48
Endocrine	26 (30.2%)	36 (41.4%)	0.37
Musculoskeletal	22 (25.6%)	29 (33.3%)	0.26
Other long-term condition	19 (22.1%)	23 (26.4%)	0.33
Respiratory repeat prescriptions (median (IQR))	6 (5–8)	4 (3–5)	<0.001
Non-respiratory repeat prescriptions (median (IQR))	7 (4–10)	7 (4–10)	0.920
Exacerbations^f,c^			
Patients with event	81 (95.3)	73 (83.9)	0.015
Exacerbations per patient year (median, IQR)	4 (2–7)	4 (1–6)	0.199
Respiratory hospitalisations^g^			
Patients with event	42 (48.8)	46 (52.9)	0.595
Respiratory admissions per patient year (median IQR)	1 (0–2)	1 (0–2)	0.568
Days in hospital per patient year (median (IQR)	0.00 (0–18.0)	1.00 (0–11.0)	0.530
Cost per patient year (median, IQR)	0.00 (0–£49010)	£2926 (0–£33649)	0.787
Respiratory clinic attendance^g^			
Patients with event	85 (98.8)	50 (57.5)	<0.001
Clinic attendance per patient year	3 (2–4)	1 (0–2)	<0.001
Cost^i^ median (IQR)	£711 (£474–£948)	£237 (0–£474)	<0.001
Respiratory Specialist Nurse home visit^g^			
Patients with event	34 (39.5%)	36 (41.4%)	0.805
Cost^i^ median (IQR)	0 (0–£49)	0 (0–£49)	0.780

^a^*n*% unless otherwise stated

Missing data:

^b^Intervention n = 1, comparator n = 8

^c^Intervention *n* = 3

^d^Interventionn = 1

^e^Intervention n = 1, comparator n = 18

^f^Data from 4 month period prior to first consultation in intervention group, and four months before 24th August 2015, in the comparator group

^g^In year prior to inclusion in the study

^h^Intervention n = 4, comparator = 12

^i^per year

The majority of patients (92.8% in the intervention group; 89.3% in the comparator group) had moderate, severe, or very severe COPD, with 80–90% MRC score 4 or 5. Patients in both groups were predominantly female and lived in areas of high socioeconomic deprivation. Despite being younger and having fewer co-morbidities, there were statistically significant differences in the distribution of FEV1 staging between intervention and usual care groups with fewer patients in the intervention group having mild FEV1 readings, and more with readings assigned as ‘very severe’. More patients in the intervention group had exacerbations in the 4-month period prior to inclusion in the study, however, exacerbations per patient year were balanced. There were fewer non-respiratory co-morbidities in the intervention group although there were a median of seven repeat prescriptions for non-respiratory causes. Hospitalisations (proportion of patients hospitalised; median number of admissions per patient and days in hospital) were comparable between groups. Patients in the intervention group were almost twice as likely to have attended a multidisciplinary out-patient appointment; the median number of attendances per patient was three times that of patients in the comparator group. Table 3 describes dispensed respiratory medicines at baseline and follow-up in both the groups: 4-months of follow-up repeat (maintenance) respiratory medicines dispensed in the period November 2015 to February 2016 for patients in the comparator group and 4 months after the first patient visit, in the intervention group. Eight patients who died during this 4-month period (four patients in the intervention group and four patients in the comparator group) were not included in maintenance prescribing data collection at follow-up.

**Table 3:** Respiratory repeat (maintenance) prescribing at baseline and follow-up

	Baseline^a^			Follow-up		
	Intervention group *n* = 88	Comparator group *n* = 87	*p-* value	Intervention (*n* = 82)	Comparator (*n* = 83)	*p*-value
Short acting beta2 agonist (SABA)	73 (84.9%)	76 (87.4%)	0.638	79 (90.8%)	77 (89.5%)	0.78
Short acting muscarinic antagonist (SAMA)	3 (3.5%)	2 (2.3%)	0.641	6 (7.0%)	1 (1.1%)	0.05
Long acting muscarinic antagonist (LAMA)	72 (83.7%)	68 (78.2%)	0.352	72 (82.8%)	71 (82.6%)	0.97
Inhaled corticosteroid (ICS)/long acting beta-adrenoreceptor agonist (LABA)	75 (87.2%)	62 (71.3)	0.010	74 (85.1%)	70 (81.4%)	0.51
LAMA/LABA	0 (0%)	1 (1.2%)	0.217	1 (1.2%)	2 (2.3%)	0.77
LABA	2 (2.3%)	3 (3.5%)	0.659	1 (1.2%)	1 (1.1%)	0.57
ICS	4 (4.6%)	6 (6.9%)	0.527	4 (4.7%)	1 (1.1%)	0.16
Mucolytic	59 (68.6%)	38 (43.7%)	0.001	62 (72.1%)	45 (51.7%)	0.006
Antihistamine	22 (25.6%)	8 (9.2%)	0.004	26 (30.2%)	7 (8.0%)	<0.001
Leukotriene receptor antagonist (LRA)	17 (19.8%)	5 (5.7%)	0.006	21 (24.4%)	4 (4.6%)	<0.001
Theophylline	20 (23.3%)	12 (13.8%)	0.109	18 (20.9%)	15 (17.2%)	0.54
Antibiotic (maintenance)	58 (67.4%)	25 (28.7%)	<0.001	60 (69.8%)	26 (29.9%)	<0.001
Prednisolone (maintenance)	29 (33.7%)	6 (6.9%)	<0.001	32 (37.2%)	14 (16.1%)	0.002
Antifungal	3 (3.5%)	0 (0.0%)	0.079	2 (2.3%)	0 (0%)	0.005
Saline (inhaled)	11 (12.8%)	6 (6.9%)	0.193	15 (17.4%)	16 (18.4%)	0.67
Benzodiazepine	15 (17.4%)	11 (12.6%)	0.377	22 (25.6%)	21 (24.1%)	0.83
Opioid (oral)	11 (12.8%)	14 (16.1%)	0.537	16 (18.6%)	23 (26.4%)	0.218

^a^At first consultation

Greater numbers of patients in the intervention group were receiving maintenance pharmacotherapy for COPD and related respiratory problems: inhaled corticosteroid (ICS)/LABA; mucolytics; antihistamines, leukotriene receptor antagonist (LRAs), and notably, antibiotics and prednisolone, with a resulting median of six respiratory medicines compared with four in the comparator group. Table 4 describes changes introduced by the pharmacist, after consulting with patients in the intervention group. The changes described in Table 4 were collected by the researcher accessing each patient’s electronic clinical record or by contacting the patient’s GP. Alterations to pharmacotherapy were common and wide-ranging, including respiratory and non-respiratory conditions. Most changes introduced by the pharmacist involved medicines: increase/decrease doses; start/stop; from one drug type to another within the same class; starting a new medicine or addressing poor inhalation technique (half of those receiving intervention, although only 8% subsequently changed their inhaler device). At least 72% of patients received at least one change to their medicines with the most common being dose changes followed by alteration of one prescribed drug to another. The pharmacist referred over half (46 (53.5%) of patients to health services in response to identified problems with co-morbid conditions. A quarter of referrals involved expediting respiratory out-patient clinic appointments, as the pharmacist responded to the patient showing signs and symptoms of clinical deterioration.

**Table 4:** Prescribing and other changes due to pharmacist intervention

	*N* (%) patients *n* = 86	*N* (%) of changes *n* = 516
Medication changes^a^		
Dose	62 (72.1)	153 (29.6)
Drug	45 (52.3)	80 (15.5)
New drug initiated	39 (45.3)	70 (13.6)
Existing drug discontinued	46 (53.5)	69 (13.4)
Inhaler technique corrected through demonstration	39 (45.3)	52 (10.1)
Oxygen prescription changed	9 (10.5)	9 (1.7)
Restart previously prescribed drug	28 (32.5)	36 (7.0)
Formulation	16 (18.6)	17 (3.3)
Repeat prescription /community pharmacy re-ordering	9 (10.5)	11 (2.1)
Change inhaler device	7 (8.1)	7 (1.4)
Dose timing	4 (4.6)	4 (0.8)
Adherence issue	3 (3.5)	4 (0.8)
Adverse drug reaction	2 (2.3)	2 (0.4)
Duration of treatment	2 (2.3)	2 (0.4)
Referrals		*n* = 127
Dual energy X-ray absorptiometry	33 (38.4)	33 (26.0)
Health services (non-respiratory)^b^	46 (53.5)	57 (44.8)
Respiratory out-patient clinic	21 (24.4)	27 (21.2)
Investigations, e.g., ECG, blood gasses, CT scan	10 (11.6)	10 (7.8)

^a^Respiratory medicines and others, e.g., azithromycin, lanzoprazole, residronate, morphine, nortryptiline. Itraconazole, gabapentin, statin

^b^For example: immunology, pulmonary rehabilitation, pain clinic, cardiology, diabetic foot check, physiotherapy.

**Fig. 2:**
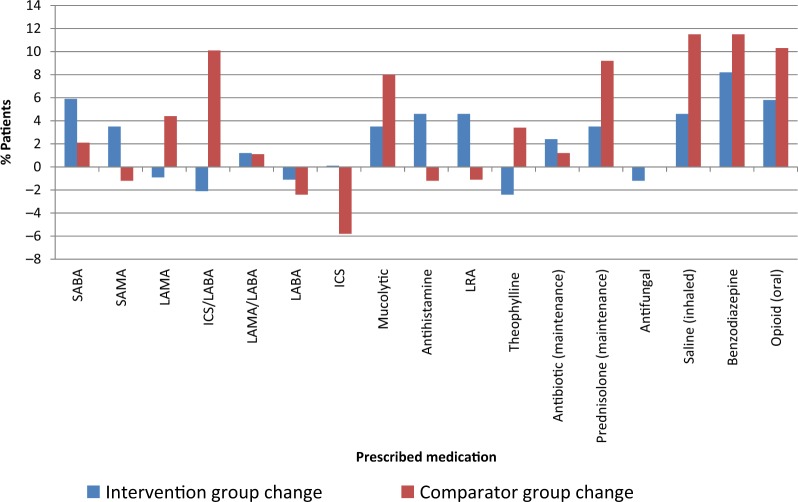
Changes in respiratory repeat (maintenance) dispensing for intervention and comparator groups from baseline to follow-up

Figure 2 shows changes in repeat dispensing for both groups from baseline to follow-up.

There were notable increases in the use of the following respiratory medicines suggesting a move towards more targeted treatment in the comparator group: ICS/LABA (from 71.3% to 81.4%); mucolytic (from 43.7% to 51.7%); prednisolone (from 6.9% to 16.1%); inhaled saline (from 6.9% to 18.4%); benzodiazepine (12.6% to 24.1%); and opioids (16.1% to 26.4%) together suggesting progression towards end stage COPD. Antihistamine and LRA prescribing, which was higher in the intervention group at baseline, increased further, from 19.8% to 24.4% and from 25.6% to 30.2%, respectively. Corresponding proportions of patients receiving LRAs and antihistamines decreased in the comparator group from baseline to follow-up (5.7% to 4.6% and from 9.2% to 8%, respectively). Maintenance antibiotic prescribing remained stable (and statistically significantly different) over time in both the groups.

**Fig. 3:**
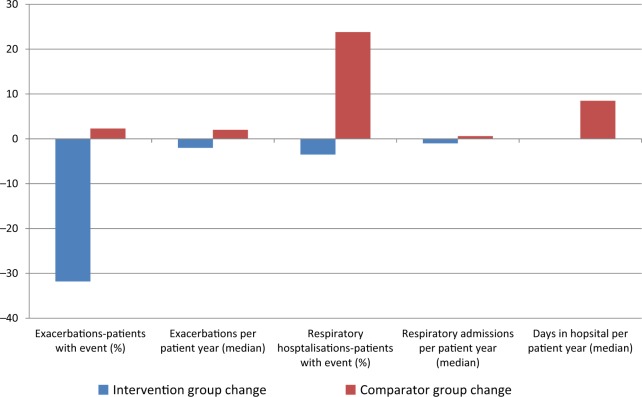
Changes in primary and secondary outcomes between baseline and follow-up for intervention and comparator groups

Table 5 compares exacerbations (primary endpoint) and respiratory hospitalisations (secondary outcomes) at follow-up and Fig. 3 shows a graphical description of the primary and secondary outcomes.

**Table 5:** Changes in primary and secondary outcomes at follow-up

Outcome	Intervention *n* = 86	Comparator group *n* = 87	*p*-value
Exacerbations^a^			
Patients with event	54 (63.5%)	75 (86.2%)	0.001
Exacerbations/patient year (median, IQR)	2 (0–6)	6 (4–10)	<0.001
Respiratory hospitalisations			
Patients with event	39 (45.3%)	66 (76.7%)	<0.001
Days in hospital/patient year (median, IQR)	0 (0–10.29)	9.48 (0–30.02)	<0.001
Respiratory admissions/patient year (median (IQR)	0 (0–2.1)	1.6 (0–3.6)	<0.001
Cost per year (mean (SD))	£32390 (£77607)	£53946 (£66748)	–

*n*% unless otherwise stated

^a^Missing values:*n* = 1

For hospitalisations and clinic visits, the mean duration of follow-up in the intervention group was 312.9 days (SD 79.5) and in the comparator group was 333.5 days (SD 68.0). Fewer patients in the intervention group had respiratory exacerbations and there were fewer exacerbations per patient year. This appeared to be due to a decrease in the intervention group over time (from 95.3% of patients at baseline to 63.5% of patients at follow-up), rather than due to an increase from baseline to follow-up in the comparator group where 83.9% at baseline increased to 86.2% at follow-up. Hospitalisations for respiratory reasons remained stable in the usual care group over time. Respiratory hospitalisations (patients with event, days in hospital and number of admissions per patient year) increased in the usual care group between baseline and follow-up, with a resulting increase in costs.

Table 6 shows attendance at respiratory clinic, and RSN attendances during the period of follow-up. Patients in the intervention group had more frequent visits to the respiratory clinic, as expected, as a result of the pharmacist intervention, which included early identification of symptom deterioration and rapid access to specialist consultant led respiratory clinics. The number of respiratory specialist nurse home visits increased over time in both groups, with the greater increase in the comparator group (from 36 (41.4%) of patients at baseline, to 84 (97%) at follow-up).

**Table 6:** Clinic, respiratory specialist nurse, and mortality during follow-up

Respiratory clinic attendance	Intervention *n* = 86	Comparator group *n* = 87	*p*-value
Patients with event (median, IQR)	70 (81.4)	50 (57.5)	<0.001
Event rate/patient year (median, IQR)	2.1 (1.0–3.3)	1.6 (0–3.2)	0.014
Cost per year (mean, SD)	£460 (£332)	£291 (£312)	−
Respiratory specialist nurse home visit			
Patients with event	44 (51%)	84 (97%)	<0.001
Event/patient (median (IQR))	1 (0–2)	3 (1–4)	<0.001
Cost (mean (SD)	£25 (£19)	£48 (£46)	−
Death from any cause			
Patients with event	14 (16.3%)	19 (21.8%)	0.53

Summing the cost of respiratory hospitalisations; clinic attendances; and RSN visits; together with the NHS salary cost of the pharmacist (£17,215); and adding the cost of consultant time (based on 1 h per week: £2000), overall there was a mean net saving of £2205 (£25 per patient per year) in the intervention group. There were no excess deaths during follow-up in either group.

## Discussion

### Main findings

A majority of patients accepted pharmacist input, which included multiple, wide-ranging changes to individual patients’ respiratory and non-respiratory medicines, and onward referral. Modest, statistically significant reductions in respiratory exacerbations, respiratory hospitalisations, and respiratory nurse home visits were observed after introducing the pharmacist intervention, in comparison with a non-randomised control group, with an increased frequency of attendances at consultant respiratory physician clinic. The intervention may have been cost saving, based on costs of staff time, clinic attendance, and hospitalisation.

### Strengths and weaknesses

As far as we are aware, pharmacist home visits for patients with COPD, where the pharmacist assesses and addresses the management of COPD and multiple morbidities, has not previously been investigated. The pharmacist collaborated with the patient’s consultant physician, and together they instigated, co-ordinated, implemented, and followed up individualised action plans, in line with recommended evidence.^33^ The intervention was delivered as planned, to patients who were already receiving care from a multidisciplinary primary and secondary care team, typical of those within the NHS in the UK. The clinical pharmacist received no specific additional respiratory therapeutics training prior to visiting patients, suggesting the intervention could be implemented more widely, at no additional cost, by clinical pharmacists who are established in general practices across the UK,^20^ but not targeting patients with COPD who live at home. The intervention had a theoretical underpinning—aiming to improve disease specific self care and prescribing for respiratory and non-respiratory conditions—and onward referral, all of which are important to help prevent exacerbations in patients with COPD living in the community.

The extent of the decrease in exacerbations in the intervention group was marked, as was the increase in exacerbations per patient year in the comparator group over time. Exacerbations and hospitalisations reduced over time and relative to the comparator group. Consultant respiratory clinic attendance increased and together with the increase in prescribing of medicines known to improve outcomes, suggests a signal of benefit, which may be due to pharmacist led case management, although further work is required to establish causality due to limitations of the methods. Outcome data collection using linkage of each patient’s unique health number to the patient’s current health records, minimised errors, and reduced the cost and risk of bias from selective outcome reporting. A similar, but less accurate approach, using algorithms to match patients’ demographic data to their health records, has been compared favourably with traditional clinical trial methods.^34,35^ Our estimation of costs did not include the cost of medicines, because of limitations in time available to the researcher. Given the importance of medicines in the study, and the cost of some of the medicines prescribed, e.g., leukotriene receptor antagonists and combination inhalers, future work should include identification and measurement of medicines costs.

Several methodological issues biased the results. The findings should be treated with caution given the significant risk of bias arising from the lack of a randomised control group, and the absence of a power calculation to inform sample size and duration of follow-up. There were statistically significant imbalances between intervention and usual care groups, in key variables at baseline (younger age; fewer co-morbidities; more antibiotic, steroid, mucolytic, antihistamine, and leukotriene receptor antagonist prescribing) all of which might be expected to confound the results by reducing exacerbations in the intervention group. However, exacerbations were more common in the intervention group than in the comparator group at baseline; which may be due to more patients in the intervention group having baseline data collected over winter months (when exacerbations are more common), compared with the comparator group where baseline data were collected for all patients from April to August. Similarly, the months of follow-up differed between intervention and comparator groups (more summer months included in the intervention group), which may also have biased the finding that exacerbation rates improved in the intervention group relative to the comparator group. Hospitalisations (number and duration) were collected over the year before and after the intervention in the active group, and 1 year before and after the mid point date in the comparator group, therefore, were not subject to the bias due to seasonal variation seen in exacerbations.

Lack of assessment of symptoms, knowledge about symptoms and management or adherence in both groups may be considered another weakness, and require to be addressed in subsequent work.

### Strengths and weaknesses in comparison to other studies

We included a modest number of patients, and a comparable duration of follow-up, to previous evaluations of pharmacist care of patients with COPD^17^ and controlled trials of complex interventions for COPD in general.^14,15,18,36^ In comparison with other studies, participants were more likely to be female; older; from more socioeconomically deprived areas; have more co-morbid conditions and polypharmacy, and more advanced respiratory symptoms.^17,18^ Our intervention breaks new ground, by focussing on COPD while also addressing polypharmacy and multiple morbidities, and involvement of a GP-based clinical pharmacist rather than a community pharmacist.^37^ Participating patients had more than double the number of prescribed medicines than previously described in a descriptive study of 78 patients with COPD,^38^ suggesting a greater associated medication burden^39^ and the utility of clinical pharmacist input to review and modify respiratory and non-respiratory medicines. A wider range of pharmacist interventions were employed, e.g., rapid referral directly into the respiratory clinic, initiation of any medicine for any condition, compared with previous pharmacist interventions that have tended to focus on COPD education, adherence, and inhaler technique.^14–18^ The link with a consultant respiratory physician contributed to patients receiving appropriate and rapid changes to their medicines and the pharmacist’s access to general practices, enabled changes to repeat and acute prescribing through conventional channels. The current study included a comparator group, as opposed to a pre-and post-study design using only an intervention group.^25,40^ Our study agrees with other studies where pharmacist intervention reduced respiratory hospitalisation^17^ and exacerbations^14^ although our outcomes were achieved through a different intervention, and in a cohort with multiple co-morbidities, which negatively influences disease expression and survival.^41,42^

In pragmatic trials involving complex intervention testing, intervention fidelity is an important consideration because the effect of the intervention depends on whether all elements are implemented as planned.^43^ The number and duration of treatment sessions delivered by the pharmacist varied according to the pharmacist’s and patient’s perceptions of length of time needed to assess and address relevant issues. The median of four per patient is considerably less than the mean of 13.7 cited in systematic reviews of previous work.^18^ The duration of each patient contact could also be important in building trust and developing a therapeutic relationship; the pharmacist spent on average, 30 min with patients in their homes at each visit. Improved outcomes are likely to be a function of the skills and knowledge of the pharmacist, rapid input from the consultant physician.

### Meaning of the study

Pharmacotherapy is one of the main approaches in the management of COPD with appropriate treatments reducing the frequency and severity of exacerbations,^2^ but polypharmacy is burdensome and associated with patients hospitalised with acute exacerbations.^44^ It is plausible that repeated pharmacist intervention to ensure optimal pharmacotherapy and minimise adverse effects, with a direct link to a consultant respiratory physician, and the patient’s GP, may lead to improved outcomes. Confounding these findings are a lack of a randomised controlled group, and different periods for follow-up data collection between groups, all of which require to be addressed before attributing causation.

Our a-priori hypothesis was that a novel model of care involving collaboration between pharmacist and consultant respiratory physician could improve outcomes. While all patients (including those with COPD) are registered with a GP, we targeted a subset of patients with severe and disabling COPD, who were already receiving secondary care input from consultant respiratory physicians at the hospital outpatient respiratory clinic. We, therefore, chose to try to enhance the existing care process between consultant respiratory physician and patient through pharmacist intervention, rather than focus on the care process between patient and GP. Our team has previously tried to enhance the care process between patient and GP, through pharmacist intervention in a previous study examining outcomes in patients with heart failure due to LVSD.^23^ This approach had limited success (albeit for a different group of patients) and the learning encouraged us to target a sicker group of patients by supporting consultants directly.

Our intervention maintained the notion of community care, by ensuring all changes agreed between pharmacist, consultant respiratory physician, and patient were communicated to the patient’s GP. The intervention was in addition to community care; it did not replace any aspect of community care.

If subsequently proven to be effective and cost effective in a pragmatic randomised controlled trial, the current fiscal climate and a shortage of GPs would make clinical pharmacist home visits an attractive proposition,^45^ in COPD where tailored pharmacotherapy and non-pharmacologic interventions, e.g., pulmonary rehabilitation, are important to maintain health.^7,46^ A novel, pharmacist, and respiratory physician collaborative intervention is feasible and may improve outcomes, and merits investigation in a randomised controlled trial.

## Methods

We examined the effect of pharmacist intervention in a non-randomised controlled pilot intervention study with a contemporaneous comparator group.

### Setting

NHS Greater Glasgow and Clyde (GG&C) Health Board is Europe’s largest health board, providing healthcare for approximately 1.15 million patients in the West of Scotland. Primary care medical services are provided free of charge through 240 general practices. General practices are independent contractors providing medical services for patients within the Health Board. Each patient is registered with one general practice where they can be seen by any of the GPs within the practice. Patients rarely chose to change practice other than for practical reasons, e.g., due to moving house. Practices have employed, permanent staff, e.g., practice nurses, and may also have visiting, temporary staff, e.g., podiatrists, who are employed by the Health Board. For the duration of the study, other than the pharmacist delivering the intervention described in this study, no respiratory specialist pharmacists were employed by or visited the practices of patients involved in the study. Secondary care services are provided through 35 hospitals, with seven specialist respiratory nurse (RSN) teams. Patients with COPD receive specialist care from consultant respiratory physicians and RSNs, in out-patient COPD clinics or during hospitalisation for respiratory reasons. In the immediate post hospital discharge period, RSNs visit patients at home.

### Inclusion criteria

Both groups were managed in the same health board, and the same regular care processes were in place in all Glasgow hospitals. The intervention group out-patient facility ran a COPD specialist clinic from which patients were recruited for the pharmacist intervention. No similar pharmacist service was run at the comparator group out-patient facility. To be eligible for inclusion patients had to have: spirometrically confirmed COPD; their name on the clinic list of the specialist respiratory team of the New Victoria or Queen Elizabeth University Hospital during March 2015; and live at home. The intervention group was identified from the New Victoria respiratory clinic list cluster and the comparator group were identified from the Queen Elizabeth University Hospital RSN cluster, to avoid contamination in this non-randomised pilot study. Both hospitals were located within NHS Greater Glasgow and Clyde and all clinical staff followed shared NHS GG&C guidelines for the management of COPD and other conditions.

### Pharmacist intervention

A general practice-based clinical pharmacist with an interest in respiratory therapeutics, worked 3 days per week, collaborating with a specialist respiratory physician, over 1 year. All patients were offered pharmacist home visits. During this first consultation, the pharmacist and the patient decided, on the basis of the pharmacist’s assessment (Appendix I) and professional judgement, whether there was merit in return visits to introduce and follow-up changes.

After collecting demographic and symptoms scores, the pharmacist discussed each of the patients’ medications, for each condition (respiratory and non-respiratory), respiratory symptoms, exacerbation frequency, and possible triggers were discussed and patients were encouraged to respond promptly to the symptoms of an exacerbation by starting oral steroids; antibiotic therapy (when sputum was purulent), and increasing the dose of inhaled therapy, in line with best practice.^7^ Positive reinforcement of the importance of medicines and symptom awareness was given at each visit. The patient’s non-respiratory medicines and co-morbid conditions were discussed and where appropriate, the pharmacist made recommendations for change to the patient’s GP using a previously established communication pathway, which results in GP acceptance and pharmacist implementation of the changes.^23^ Patients were prospectively screened for risk of osteoporosis using FRAX score and referred for bone mineral density dependant on this result.

The pharmacist and patient agreed an individualised respiratory management plan and medicines changes or onward referrals were implemented by the pharmacist with consultant respiratory physician input to respiratory matters. Scheduled follow-up visit dates were agreed, and the pharmacist implemented and co-ordinated communication of all of the changes, by contacting all relevant stakeholders: the patient’s GP, community pharmacist and any services to which the patient was referred, e.g., dual energy X-ray absorptiometry (DEXA); smoking cessation support; or referral for pulmonary rehabilitation. Specific therapeutic approaches included targeted or long-term antibiotics in those found to have bacterial colonisation; high-dose inhaled corticosteroids, antihistamines, or leukotriene receptor antagonists in patients with features suggesting COPD with asthma; treatment of fungal infection; or palliative care. Patients were asked if and how they took their medicines, and individualised support provided if necessary, e.g., simplification of dose regimens therefore, assessing and addressing adherence was not a common feature of the intervention received by each patient, whereas, positive reinforcement of the importance of specific parts of the patient’s therapeutic regimen, were covered at each visit. Inhaler technique was corrected where necessary by demonstration using placebos.

### Usual care

All patients (including those who received the pharmacist’s intervention) continued to receive usual care from their GP and multidisciplinary specialist respiratory team (consultant respiratory physician and RSN). Comparator group patients received no clinical pharmacist input throughout the study period.

### Consent

The NHS Greater Glasgow and Clyde Respiratory Managed Clinical Network and NHS Greater Glasgow and Clyde Ethics screener approved the project as a new service evaluation; as such, written consent was not required or requested from participants.

### Measures

#### Data collection

In Scotland, and the rest of the UK, the patient’s GP has an electronic record containing all of the patient’s clinical information, including details of secondary care contacts and prescribed medicines. Data on dispensed medicines are not available within GP records, because data on dispensed medicines are captured in community pharmacies, where the medicines are dispensed to patients on receipt of a prescription. Data on dispensed medicines captured in community pharmacies are sent electronically (automatically linked to the patient’s CHI number (a ten digit unique identifier based on date of birth, assigned to each patient, by NHS Scotland), to a central electronic store (Prescribing Information System, PIS, part of Information Services Division, NHS Scotland) and made available to NHS Staff on demand, e.g., for service evaluation. While the information contained in GP records can only be accessed in the practice and not remotely, some of the data contained in the GP records (e.g., hospitalisations; out-patient clinic attendance, and specialist respiratory nurse domiciliary visits) can also be accessed remotely through a clinical record system called Clinical Portal. Again, these data are accessible for individual patients, using the patient’s CHI number. In the study, we made use of all of these systems for data collection at baseline and follow-up.

Baseline demographic and clinical data were extracted from hospital and GP-based electronic case records. COPD assessment test (CAT) scores were only collected in the Intervention group, by the pharmacist, at the first consultation. Routine clinical data sources used to access information on comparator group patients did not include CAT scores.

All medicines were obtained by the patient from their community pharmacy. Given the unreliability of questionnaires as measures of assessing adherence,^47^ we used community pharmacy dispensed prescribing records as our measure of adherence. Dispensed prescriptions (baseline and follow-up) were collected using PIS, which captured drug names; doses; dispensing date; quantities; and formulations dispensed in community pharmacies in Scotland.^48^ Two types of prescription can be ascertained from dispensed data: repeat (maintenance, where the PIS record shows the patient has collected enough of their medicine to enable daily dosing) and acute (episodic, short courses of medicines including treatment for exacerbations, inferred from the date and quantity of collected medicine on the PIS record). A supply of medicine was categorised as repeat (maintenance) if the patient’s linked electronic dispensing record showed at least 4 consecutive months of enough dispensed medicines to enable daily dosing. The supply of medicine was categorised as acute when the medicine was dispensed as one or more short courses because this meant the patient did not have sufficient supplies of medicine to take it every day over a 4-month period. Given the importance of primary care management of COPD to prevent exacerbations requiring hospitalisation, we selected the number of primary care exacerbations as our a-priori primary outcome. An exacerbation included short courses of antibiotics (5–14 day course of penicillin, macrolide, or doxycycline either as a change to antibiotic class in patients already receiving maintenance antibiotics, or initiation in a patient not previously receiving one) with or without concomitant high-dose steroid (5–14 day course of prednisolone at a dose greater than 10 mg or a higher than maintenance dose in patients already receiving maintenance steroids) or high-dose steroid alone.^7^

In the intervention group, exacerbations at baseline were obtained by extracting PIS data over the 4-month period prior to each patient’s first contact with the pharmacist. In the comparator group, PIS exacerbation data were also extracted over a 4-month period—prior to the 24th August 2015 (the date of the mid point of consultations in the intervention group).

Follow-up data on the number of exacerbations were collected over the 4-month period November 2015 to February 2016 for patients in the comparator group, and in the 4-month period from the date of first consultation for patients in the intervention group.

Hospitalisations, multidisciplinary out-patient clinic attendance and nurse domiciliary visits, were collected from the electronic clinical management system used to record the date and cause of hospital admissions (Clinical Portal). At baseline, these were collected over 1 year prior to the date of first consultation in the intervention group, and from 24th August 2014 to 24th August 2015 in the usual care group. At follow-up, hospitalisations, multidisciplinary respiratory out-patient attendances and respiratory nurse specialist home visits were collected over 1 year for all patients: 1 year from the date of each first consultation in patients in the intervention group, and 1 year from the 24th August 2015 for patients in the comparator group. In both groups, censoring took place on the date of death.

#### Costs

The cost of admissions, multidisciplinary out-patient clinic visits, and respiratory specialist nurse home visits were obtained from ISD Scotland, and summed over the year before and year after each patient’s final visit (in the intervention group) or in the year before and year after the index date of 24th August 2015 (in usual care). Costs were obtained by multiplying the net cost of an inpatient bed day, out-patient attendance or RSN, by the number of days in hospital or number of clinic attendances or the number of specialist nurse visits, respectively. Pharmacist and RSN costs were taken from Agenda for Change pay scales and consultant physician time were obtained directly from NHS GG&C. Medicines costs were not included in our analysis.

### Statistical analysis

Continuous variables are described using mean (SD), median, inter-quartile range (IQR), and range as appropriate. Categorical variables are described using the number and percentage falling into each category reported, with percentages calculated relative to the number of non-missing. The number missing was reported for all variables. Differences between the pharmacist intervention and comparator group in terms of the observed variables were tested using two sample *t*-tests or Mann–Whitney tests for continuous data, depending on the distributions. Categorical variables were analysed using Chi-squared tests or Fisher’s tests as appropriate. For all analyses, a *p*-value < 0.05 was considered to indicate statistical significance; *p*-values are shown for reference. Statistical analyses were carried out using MINITAB version 16.

## Electronic supplementary material

Appendix I

## Data Availability

The data that support the findings of this study are available from the authors upon reasonable request and with permission of NHS Greater Glasgow and Clyde.

## Electronic supplementary material

**Supplementary information** accompanies the paper on the *npj Primary Care Respiratory Medicine* website (10.1038/s41533-018-0105-7).

## References

[1] Lozano R (2012). Global and regional mortality from 235 causes of death for 20 age groups in 1990 and 2010: a systematic analysis for the Global Burden of Disease Study 2010. Lancet.

[2] *Global Strategy for the Diagnosis, Management and Prevention of COPD, Global Initiative for Chronic Obstructive Lung Disease (GOLD)*. 2016. http://goldcopd.org/. Accessed Jan 2018.

[3] Mathers CD, Loncar D (2006). Projections of global mortality and burden of disease from 2002 to 2030. PLoS Med..

[4] Barnes PJ, Celli BR (2009). Systemic manifestations and comorbidities of COPD. Eur. Respir. J..

[5] American Thoracic Society Foundation. *The Global Burden of Lung Disease*. (2014). http://foundation.thoracic.org/news/global-burden.php (Accessed Jan 2018).

[6] McAllister DA, Morling JR, Fischbacher CM, MacNee W, Wild SH (2013). Socioeconomic deprivation increases the effect of winter on admissions to hospital with COPD: retrospective analysis of 10 years of national hospitalisation data. Prim. Care. Respir. J..

[7] National Institute for Health and Clinical Excellence. *Clinical Guideline 101; Chronic Obstructive Pulmonary Disease in over 16s: Diagnosis and Management*. (2010). https://www.nice.org.uk/guidance/CG101. 31211541

[8] Marsden E, Cubbin I, McAlavey A (2009). An investigation into how poor compliance traditionally associated with corticosteroid therapy in asthma and chronic obstructive pulmonary disease can be improved to enhance long-term management and patient care. Int. J. Pharm. Pract..

[9] Rand CS (2005). Patient adherence with COPD therapy. Eur. Respir. Rev..

[10] Hand H, Bradley C (2009). Health beliefs of adults with asthma: toward an understanding of the difference between symptomatic and preventative use of inhaler treatment. J. Asthma.

[11] Vestbo J (2009). Adherence to inhaled therapy, mortality and hospital admission in COPD. Thorax.

[12] Cochrane GM (1992). Therapeutic compliance in asthma; its magnitude and implications. Eur. Respir. J..

[13] Black PN, McDonald CF (2009). Interventions to reduce the frequency of exacerbations of chronic obstructive pulmonary disease. Postgrad. Med. J..

[14] Tommelein E (2013). Effectiveness of pharmaceutical care for patients with chronic obstructive pulmonary disease (PHARMACOP): a randomised controlled trial. Br. J. Clin. Pharmacol..

[15] Khdour MR, Kidney JC, Smyth BM, McElnay JC (2009). Clinical pharmacy-led disease and medicine management programme for patients with COPD. Br. J. Clin. Pharmacol..

[16] Jarab AS, Alqudah SG, Khdour M, Shamssain M, Mukattash TL (2012). Impact of pharmaceutical care on health outcomes in patients with COPD. Int. J. Clin. Pharm..

[17] Zhong H, Ni XJ, Cui M, Liu XY (2014). Evaluation of pharmacist care for patients with chronic obstructive pulmonary disease: a systematic review and meta-analysis. Int. J. Clin. Pharm..

[18] Dickens C (2014). Complex interventions that reduce urgent care use in COPD: A systematic review with meta regression. Resp. Med..

[19] Anon. Royal Pharmaceutical Society. *Pharmacists and GP Surgeries: Policy topic*. 12th Oct 2016. https://www.rpharms.com/making-a-difference/policy-a-z/pharmacists-and-gp-surgeries (Accessed 8th February 2018).

[20] Anon. *RCGP and RPS Policy Statement on GP Practice based pharmacists*. Royal Pharmaceutical Society/Royal College of General Practitioners. Feb 2015 https://www.rpharms.com/Portals/0/RPS%20document%20library/Open%20access/Policy%20statements/rcgp-joint-statement-for-pharmacists-in-gp-surgeries.pdf (Accessed Feb 2018).

[21] Mackie CA, Lawson DH, Campbell A, Maclaren AG, Waigh R (1999). A randomised controlled trial of medication review in patients receiving polypharmacy in general practice. Pharm. J..

[22] Maclaren AG, Mackie CA, Lowrie R, Tennant S (2003). Medication review: Recruitment and accreditation of a cohort of community pharmacists. Int. J. Pharm. Pract..

[23] Lowrie R (2011). Pharmacist intervention in primary care to improve outcomes in patients with left ventricular systolic dysfunction. Eur. Heart J..

[24] Lowrie R, Morrison J, McConnachie A (2010). A cluster randomised controlled trial of pharmacist led Statin Outreach Support (SOS) in primary care: Design and baseline characteristics. Cont. Clin. Trials.

[25] Wright D (2015). An evaluation of a multi-site community pharmacy-based chronic obstructive pulmonary disease support service. Int. J. Pharm. Pract..

[26] Crockett A (2002). Managing Chronic Obstructive Pulmonary Disease in Primary Care.

[27] Lowrie R (2017). Incentivised chronic disease management and the inverse equity hypothesis: findings from a longitudinal analysis of Scottish Primary Care practice level data. BMC Med..

[28] Kontopantelis E (2016). Associations between exemption and survival outcomes in the UK’s primary care pay-for performance programme: a retrospective cohort study. BMJ Qual. Saf..

[29] Postma DS (1999). Home treatment of COPD exacerbations. Thorax.

[30] National Institute for Health and Clinical Excellence. *Clinical Guideline 101; Chronic Obstructive Pulmonary Disease in over 16s: Diagnosis and Management* (2010). https://www.nice.org.uk/guidance/cg101. Accessed October 2016.

[31] Divo M (2012). Comorbidities and risk of mortality in patients with chronic obstructive pulmonary disease. Am. J. Resp. Crit. Care Med..

[32] NHS Institute for innovation and improvement. (2010). Directory of Ambulatory Emergency Care for Adults.

[33] Turnock AC, Walters EH, Walters JA, Wood-Baker R (2005). Action plans for chronic obstructive pulmonary disease. Cochrane Database Syst. Rev..

[34] Ford I (1995). Computerised record linkage: compared with traditional patient follow-up methods in clinical trials and illustrated in a prospective epidemiological study. The West of Scotland Coronary Prevention Study Group. J. Clin. Epidemiol..

[35] Ford I (2007). Long-term follow-up of the West of Scotland Coronary Prevention Study. N. Engl. J. Med..

[36] Tsai CL, Griswold SK, Clark S, Camargo CA (2007). Factors associated with frequency of emergency Department visits for chronic obstructive pulmonary disease exacerbation. J. Gen. Intern. Med..

[37] van Molen T (2017). Optimizing identification and management of COPD patients-reviewing the role of the community pharmacist. Br. J. Clin. Pharmacol..

[38] Dolce JJ, Crisp C, Manzella B (1991). Medication adherence patterns in chronic obstructive pulmonary disease. Chest.

[39] Negewo NA (2017). Treatment burden, clinical outcomes, and co-morbidities in COPD: an examination of the utility of medication regimen complexity index in COPD. Int. J. Copd..

[40] van Boven JF, Stuurman-Bieze AG, Hiddink EG, Postma MJ (2016). Effects of targeting disease and medication management interventions towards patients with COPD. Curr. Med. Res. Opin..

[41] Vanfleteren LEGW, Spruit MA, Wouters EFM, Franssen FME (2016). Management of chronic obstructive pulmonary disease beyond the lungs. Lancet Respir. Med..

[42] Decramer M, Janssens W (2013). Chronic obstructive pulmonary disease and comorbidities. Lancet Respir. Med..

[43] Bellg AJ (2004). Enhancing treatment fidelity in health behaviour change studies: best practices and recommendations from the NIH Behaviour Change Consortium. Health Psych..

[44] Diez-Manglano J (2014). Polypharmacy in patients hospitalised for acure exacerbation of COPD. Eur. Respir. J..

[45] Twaddell I. Train non-medical staff to solve GP workforce crisis, employers told. *Pulse* (17th May 2016). http://www.pulsetoday.co.uk/home/finance-and-practice-life-news/train-non-medical-staff-to-solve-gp-workforce-crisis-employers-told/20031859.article.

[46] Chong J, Karner C, Poole P (2012). Tiotropium versus long acting beta agonists for stable chronic obstructive pulomonary disease. Coch. Database Syst. Rev..

[47] Osterberg L, Blaschke T (2005). Adherence to medication. N. Engl. J. Med..

[48] Prescribing Information System. *Administrative Data Liaison System*. http://www.adls.ac.uk/nhs-scotland/prescribing-information-system/?detail (Accessed 2018).

