# Supplementary material for: A non-randomised controlled pilot study of clinical pharmacist collaborative intervention for community dwelling patients with COPD
Source: NPJ Prim Care Respir Med. 2018 Oct 10;28:38. doi: 10.1038/s41533-018-0105-7 (PMC6180130; doi:10.1038/s41533-018-0105-7)
Supplement: Supplementary file 1 — Appendix I [file 41533_2018_105_MOESM1_ESM.docx]

**Appendix I**

**COPD pro-forma for pharmacist home visit**

| **Name** | | | | | | | | | | | **CHI**  ( ) | | | | | | | | | **Audit code** | |
| --- | --- | --- | --- | --- | --- | --- | --- | --- | --- | --- | --- | --- | --- | --- | --- | --- | --- | --- | --- | --- | --- |
| **Address** | | | | | | | | | | | | | | | **Phone** | | | | | | |
| **GP:** | | | | | | **Pharmacy:** | | | | | | | | | **First visit date:** | | | | | | |
| **Social:** | | | | | | | | | | | | | | | | | | | | | |
|  | | | | | | | | | | | | | | | | | | | | | |
| **Resp diagnosis / investigations** | | | | | | | | | | | | | | | | **Date** | | | | | |
| COPD | | | | | | | | | | | | | | | |  | | | | | |
|  | | | | | | | | | | | | | | | |  | | | | | |
|  | | | | | | | | | | | | | | | | | | | | | |
|  | | | | | | | | | | | | | | | | | | | | | |
| **Last clinic** | | | **Attended** | | | | | **Prev clinic** | | | | | **Attended** | | | | | | | | |
|  | | |  | | | | |  | | | | |  | | | | | | | | |
| **Next clinic date** | | | | | | | | | | | | | | | | Mon / Fri | | | | | |
|  | | | | | | | | | | | | | | | | | | | | | |
| **Smoker?** | | | | | | | | **BMI** | | | | | | | | | | | | | |
| **Renal function:** | | | | | | | | **Liver function:** | | | | | | | | | | | | | |
| **Last PFTs** | | | | | | | | **Sats** | | | | | | | | | | | | | |
| **FEV_1_ %:**  % | | | **FVC %:** % | | | | | **Ratio**: % | | | | | | | | | **reversibility:** | | | | |
|  | | | | | | | | | | | | | | | | | | | | | |
| **Respiratory and other therapy** | | | | | | | | | | | | | | | | | | | | | |
| **Group** | **Drug, form & str** | | | | **Dose** | | **Start** | | **Compliance** | | | **effect** | | | | | | **s/e** | | | **Inh tech etc.** |
|  |  | | | |  | |  | |  | | |  | | | | | |  | | |  |
|  |  | | | |  | |  | |  | | |  | | | | | |  | | |  |
|  |  | | | |  | |  | |  | | |  | | | | | |  | | |  |
|  |  | | | |  | |  | |  | | |  | | | | | |  | | |  |
|  |  | | | |  | |  | |  | | |  | | | | | |  | | |  |
|  |  | | | |  | |  | |  | | |  | | | | | |  | | |  |
|  |  | | | |  | |  | |  | | |  | | | | | |  | | |  |
|  |  | | | |  | |  | |  | | |  | | | | | |  | | |  |
|  |  | | | |  | |  | |  | | |  | | | | | |  | | |  |
|  | | | | | | | | | | | | | | | | | | | | | |
| **MRC** | | | |  | | | | | | **CAT** | | | | | | | | |  | | |
| **PHQ-9** | | | |  | | | | | | **How difficult?** | | | | | | | | |  | | |
| **FRAX (MO#)** | | | |  | | | | | | **NOGG** | | | | | | | | |  | | |
|  | | | | | | | | | | | | | | | | | | | | | |
| **Exacerbations in last 6 months** | | | | | | | | | | **Exacerbations since 1^st^ visit** | | | | | | | | | | | |
| **Date** | | **Details** | | | | | | | | **Date** | | | | **Details** | | | | | | | |
